# “*We might not have been in hospital, but we were frontline workers in the community*”: a qualitative study exploring unmet need and local community-based responses for marginalised groups in Greater Manchester during the COVID-19 pandemic

**DOI:** 10.1186/s12913-024-10921-4

**Published:** 2024-05-13

**Authors:** Stephanie Gillibrand, Ruth Watkinson, Melissa Surgey, Basma Issa, Caroline Sanders

**Affiliations:** 1 Centre for Primary Care and Health Services Research, University of Manchester, Greater Manchester, England, UK; 2 NIHR Applied Research Collaboration for Greater Manchester, Greater Manchester, England, UK; 3 Independent (public contributor), Greater Manchester, England, UK; 4 Greater Manchester Patient Safety Research Centre, University of Manchester, Greater Manchester, England, UK

**Keywords:** VCFSEs, Community, Marginalised groups

## Abstract

**Background:**

The response to the COVID-19 pandemic saw a significant increase in demand for the voluntary, community, faith and social enterprise (VCFSE) sector to provide support to local communities. In Greater Manchester (GM), the VCFSE sector and informal networks provided health and wellbeing support in multiple ways, culminating in its crucial supportive role in the provision of the COVID-19 vaccination rollout across the GM city region. However, the support provided by the VCFSE sector during the pandemic remains under-recognised. The aims of the study were to: understand the views and experiences of marginalised communities in GM during the COVID-19 pandemic; explore how community engagement initiatives played a role during the pandemic and vaccine rollout; assess what can be learnt from the work of key stakeholders (community members, VCFSEs, health-system stakeholders) for future health research and service delivery.

**Methods:**

The co-designed study utilised a participatory approach throughout and was co-produced with a Community Research Advisory Group (CRAG). Focus groups and semi-structured interviews were conducted remotely between September-November 2021, with 35 participants from local marginalised communities, health and care system stakeholders and VCFSE representatives. Thematic framework analysis was used to analyse the data.

**Results:**

Local communities in GM were not supported sufficiently by mainstream services during the course of the COVID-19 pandemic, resulting in increased pressure onto the VCFSE sector to respond to local communities’ need. Community-based approaches were deemed crucial to the success of the vaccination drive and in providing support to local communities more generally during the pandemic, whereby such approaches were in a unique position to reach members of diverse communities to boost uptake of the vaccine. Despite this, the support delivered by the VCFSE sector remains under-recognised and under-valued by the health system and decision-makers.

**Conclusions:**

A number of challenges associated with collaborative working were experienced by the VSCE sector and health system in delivering the vaccination programme in partnership with the VCFSE sector. There is a need to create a broader, more inclusive health system which allows and promotes inter-sectoral working. Flexibility and adaptability in ongoing and future service delivery should be championed for greater cross-sector working.

**Supplementary Information:**

The online version contains supplementary material available at 10.1186/s12913-024-10921-4.

## Background

The response to the COVID-19 pandemic saw a significant increase in demand for the voluntary, community, faith and social enterprise (VCFSE) sector to provide support to local communities [1, 2]. The role of communities was seen as crucial to supporting the pandemic response, to better mobilise public health pandemic responses and supportive health services [3]. VCFSE organisations nationally had to quickly mobilise to adapt their service offer to meet increased demand, new gaps in service provision and deliver services in different ways to address the challenges faced by local communities. These included loss of income and financial hardship, closure of schools and childcare, increased social isolation, digital exclusion, and increased mental health issues [4]. However, previous research has concluded that support provided by the voluntary sector during the pandemic has been under-recognised [5]. Some authors have explored the role that VCFSEs played at the national level, in supporting communities during the pandemic [4–6]. Yet, whilst it is well-known that tens of thousands of UK volunteers supported local vaccine delivery [7], no existing academic literature has explored the role of VCFSEs in supporting the vaccination rollout.

We focus on Greater Manchester (GM), where increased support from VCFSE organisations, including smaller, community-based networks, responded to increased demand from local communities and the NHS to provide key health and wellbeing-related services, including food and care packages for clinically vulnerable households, food bank services, support for people experiencing homelessness, mental health and domestic violence services and support to local community organisations [8]. This support culminated in the sector’s supportive role in the delivery of the COVID-19 vaccination rollout, in response to the need for mass immunisation across the region.

Over the last decade, the English health and care system has been evolving to integrate health and social care. A key focus is building closer working relationships between the NHS, local authorities and other providers– including the VCFSE sector– to deliver joined up care for communities [9, 10]. To aid integration, a new model for organising health and care on different geographical footprints has been developed: Integrated Care Systems (ICSs), place-based partnerships and neighbourhood models. These collaborative partnerships bring together existing health and care organisations to coordinate health and care planning and delivery in a more integrated way and include councils, NHS provider trusts, Primary Care Networks, GP federations and health and care commissioners [11]. These new geographically-based partnerships have an emphasis on collaborative working beyond traditional health and care partners. This includes acknowledging the role that VCFSE organisations can have in supporting wider population wellbeing, particularly as part of multi-disciplinary neighbourhood teams embedded in local communities [12]. National guidance on the development of ICSs and place-based partnerships strongly encourages health and care leaders to include VCFSE organisations in partnership arrangements and embed them into service delivery [12]. In GM, the partnership working approach pre-dates the formal mandating of ICSs, with a combined authority which brings together the ten local authorities and an association of Clinical Commissioning Groups (CCGs) which represented health commissioners, and a VCFSE umbrella group which also operates as a joint venture to represent the sector’s interests at a GM level^1^. However, reorganisation to the ICS system may present new local challenges for the VCFSE sector to find a meaningful ‘seat at the table’. That withstanding, the COVID-19 pandemic coincided with the development of ICSs and place-based partnerships as arguably one of the earliest and most intense tests of partnership working across health and care organisations within the current policy landscape.

Here, we present findings from a co-designed qualitative research project, drawing on insights from 35 participants, including members of diverse communities in GM, VCFSE participants, and key decision-making health and care system stakeholders. The aims of the study were to: understand the views and experiences of marginalised communities in GM during the COVID-19 pandemic; explore how community engagement initiatives played a role during the pandemic and vaccine rollout; assess what can be learnt from the work of key stakeholders (including community members, VCFSEs, health and care system stakeholders) for future health research and service delivery. The rationale for the study developed from a related piece of work assessing inequalities in the COVID-19 vaccine uptake in GM [13]. At that time, there was little research on the experiences of under-served communities during the pandemic. As such, the public and stakeholder engagement for the related project identified a need for a qualitative workstream to explore more fully the drivers behind and context surrounding the vaccination programme in GM, centring also local communities’ experiences during the pandemic (explored in a related paper [14]).

In this paper, we examine the role the VCFSE sector played in supporting unmet needs for marginalised groups in GM during the COVID-19 pandemic and as part of the rapid rollout of the COVID-19 vaccination programme. We consider the opportunities and barriers that may influence the full integration of the VCFSE sector into health and care services in the future. This paper provides additional evidence around the role of local community-led support in the context of identified unmet needs from marginalised local communities. Whilst focused on GM, it provides an exemplar of the role of VCFSEs and community networks during the pandemic, with relevant learning for other regions and international settings with place-based partnerships.

## Methods

### Study design

The study utilised a participatory approach throughout and was co-designed and co-produced with a diverse Community Research Advisory Group (CRAG). The CRAG were members of local community groups who were disproportionately impacted by the COVID-19 pandemic, including one member who is a co-author on this paper. This included members of three VCFSE organisations working with specific ethnic minority communities including Caribbean and African, South Asian and Syrian communities.

CRAG members acted as champions for the research, supporting design of appropriate information and fostering connections for recruitment via their existing community networks. The strong partnerships built through our approach were crucial to enabling a sense of trust and legitimacy for the research amongst underserved communities invited to participate.

Interviews and focus groups took place between September-November 2021 and sought to explore: the context surrounding the rollout of the vaccination programme; key aspects of support delivered as part of the vaccination programme; the use of localised approaches to support vaccine delivery including engagement initiatives, as well as broader community-level responses to the COVID-19 pandemic; perceptions around barriers to vaccine uptake^2^; experiences of local communities (including healthcare) during the pandemic^3^. During the data collection period, national pandemic restrictions were largely lifted with no restrictions on social distancing or limits to gatherings, and all public venues reopened. A self-isolation period of 10 days after a positive COVID-19 test remained a legal requirement, but self-isolation after contact with a positive case was not required if fully vaccinated [15]. By July 2021, every UK adult had been offered their first dose of the COVID-19 vaccine, with every adult offered both doses by mid-September 2021 [16]. By early September 2021, more than 92 million doses had been administered in the UK [15].

Interviews and focus groups were conducted by one member of the research team (SG) and were conducted remotely due to the pandemic, via Zoom and telephone calls. The limitations of undertaking remote qualitative research interviews are acknowledged in academic literature, including potential restrictions to expressing compassion and assessing the participant’s environment [17, 18]. However, given the remaining prevalence of COVID-19 at the time of interview, it was judged that the ensuing risk posed by COVID-19 to both researchers and participants outweighed the potential drawbacks. Nevertheless, participants were offered face-to-face options if they were unable to participate remotely to maximise inclusion (although no participants chose to participate face-to-face).

Interviews and focus groups were audio recorded with an encrypted recorder and transcribed by a professional transcription service. Informed written consent to participate was taken prior to the interviews and focus groups. The average length of the interviews was 34 min and average length of the focus groups was 99 min. Two focus groups were co-facilitated by a CRAG member, a member of the local community who works for a mental health charity that supports local South Asian communities, who also provided translation support. In respect to authors positionality, coauthors SG, RW, MS and CS are university researchers in academic roles and had prior links to the CRAG members via a wider community forum (co-ordinated by the NIHR funded Applied Research Collaboration for Greater Manchester). The wider group met regularly to discuss and share learning regarding community experiences, community action and related research during the pandemic. BI is a member of the CRAG and a member of a local Syrian community.

### Sampling & recruitment

The sampling strategy for community participants centred around groups that had been disproportionately affected by the COVID-19 pandemic in England, including ethnic minority groups, young adults, and those with long-term physical and mental health conditions. VCFSE participants included community and religious leaders, members of local community VCFSE organisations and smaller, informal community networks and groups from local communities. Health and care system stakeholders included local council workers and health and care system stakeholders (e.g. those organising the vaccination response in CCGs and GP Federations). Characteristics of the sample are provided in Table 1. Overall, the study achieved a diverse sample of participants on the basis of gender and ethnicity.

A combination of purposive and snowballing sampling was used to recruit via pre-established links and connections to community networks and stakeholders to ensure the inclusion of specific seldom-heard groups. For example, members of African and Caribbean communities were recruited via a charity which supports the health of these groups, and members of South Asian communities were recruited via a mental health charity.

Quotes are described by respondent type (community member, VCFSE participant, health and care system stakeholder) and participant identifier number to maintain anonymity whilst providing important contextual detail.

**Table 1:** Sample characteristics (community residents, community leaders, health and care system stakeholder participants *n* = 35)

	Community resident participants (*n* = 24)	Community leaders (*n* = 6)	health and care system stakeholders (*n* = 5)
**Gender**			
Male	7	3	1
Female	17	2	4
Prefer not to say	0	1	0
**Age**			
18–24	6	0	0
30–39	2	1	1
40–49	4	3	2
50–59	3	2	1
55–64	2	0	1
60–69	3	0	0
70–79	3	0	0
80+	1	0	0
**Ethnicity**			
African/African British	1	0	0
Arab	0	2	0
Bangladeshi/Bangladeshi British	1	0	0
Indian/Indian British	2	0	0
Caribbean/Caribbean British	2	0	0
Chinese	1	0	0
Jewish	0	2	0
Kashmiri	1	0	0
Pakistani/Pakistani British	6	0	0
White English, Welsh, Scottish, Northern Irish or British	10	2	5
**Greater Manchester localities**			
Bolton	1	0	1
Bury	0	0	1
Manchester	7	2	0
Rochdale	7	0	1
Salford	4	3	2
Stockport	1	0	0
Tameside	1	0	0
Trafford	1	1	0
Other	2	0	0
**Total**	**24**	**6**	**5**

### Data analysis

We analysed the data using an adapted framework approach [19]. We adopted a framework approach to analysis as this is viewed as a helpful method when working within large multidisciplinary teams or when not all members of the team have experience of qualitative data analysis, as was the case within our team. This structured thematic approach is also considered valuable when handling large volumes of data [20, 21] and was found to be a helpful way to present, discuss and refine the themes within the research team and CRAG meetings. We created an initial list of themes from coding four transcripts, and discussions with CRAG members: personal or family experiences/stories; work/education experiences; racism and racialised experiences; trust and mistrust; fear and anxiety; value of community/community approaches; access to services including healthcare; operational and logistical factors around vaccine rollout; communication and (mis)information. We used this set of themes and sub themes to code the remaining transcripts, including further inductively generated codes as analysis progressed, regularly discussing within the team.

We shared transcript coding amongst the study team, with one team member responsible for collating coded transcripts into a charting framework of themes/subthemes with illustrative transcript extracts. The themes were refined throughout the analysis period (November 2021-March 2022) with the research team and CRAG and were sense-checked with CRAG members and the wider study team, to synthesise a final iteration of the themes and sub-themes (see supplementary material). We present findings related to five overarching themes: (1) unmet needs of local communities during the pandemic: inaccessible care and distrust; (2) community-led approaches: social support and leadership to support services; (3) community led support to COVID-19 vaccination delivery; (4) operational and logistical barriers to community-based pandemic responses: challenges faced by the voluntary and community sector; (5) learning from the pandemic response in GM: trust building and harnessing community assets. Themes are discussed in more detail below.

### Ethical approval

This study was approved by University of Manchester Ethics Committee (Proportionate University Research Ethics Committee) 24/06/21. Ref 2021-11646-19665.

## Results

### Unmet needs of local communities during the pandemic: inaccessible care and distrust

The COVID-19 pandemic brought an unprecedented shift in the way NHS services could function due to social distancing and lockdown measures. Pressures included unprecedented demand on hospital capacity and infection control measures (within hospitals and across the NHS) which reduced workforce capacity. There were also staff shortages due to high levels of COVID-19 infection amongst NHS staff, and shortages in non-acute capacity due to staff re-deployment [22, 23]. In an effort to reduce pressure on the NHS, the policy mantra “Protect the NHS” was coined as a keynote slogan from the early stages of the pandemic [24].

It is within this context that many community participants raised (spontaneously) that there was a general inability to access health services during the pandemic, including GP and specialist services.

when I tried to contact my doctor’s surgery I was on the call for over an hour, number 20, number 15. Then by the time I’m under ten I get cut off. And it happened continuously. I just couldn’t get through and I just gave up really…now it’s like a phone consultation before you can even go and see someone, and even for that you’re waiting two, three weeks. (1029, VCFSE participant)

This resulted in frustration amongst some community participants, who questioned the logic of “protecting the NHS”, seemingly at the expense of their health-related needs. This led to sentiments that other health needs were de-prioritised by decision-makers during the pandemic. It was felt that this logic was counter-productive and fell short of the principles of protecting the most vulnerable.

We were like it just didn’t matter, it could have been much more serious than just a cough or a cold, [] but the help was just not there” (1028, community participant).

what about people who actually need to see a doctor so the very vulnerable ones that we’re supposed to be protecting. Yes, we’re protecting the NHS, I understand that, I said, but we’ve also got to protect all those vulnerable people that are out there that are actually isolated (1011, community participant).

Community participants described their fear of accessing healthcare service because of potential risks of catching the virus in these settings, and fear of insufficient care due to well-publicised pressures in NHS settings. Some VCFSE participants noted that the widely publicised pressures faced by the NHS, and heightened media and political attention around COVID-19 cases in health settings led to fear and anxiety^4^.

I didn’t go to the hospital because I was scared shitless whether I was going to come out alive from hospital.” (1023, community participant).

…the number of people who didn’t access services when they should have done… They were either terrified they were going to go into hospital and catch COVID straightaway and die, or they were terrified that they were taking [the hospital space] away from someone else (2003, VCFSE participant).

Overall, this led to a strong sense that mainstream services were not supporting the needs of local communities. This was especially felt for those requiring specialist services (e.g. mental health or secondary services), and for those who had faced intersecting inequalities, such as health issues, language and digital/IT barriers, and newly settled refugees and immigrants.

### Community-led approaches: social support and leadership to support services

As a consequence of this unmet need, VCFSE and community participants identified that local communities themselves increased activities to provide community support. Participants felt strongly that this increased support provided by the VCFSE sector and community networks remains under-recognised and under-valued by the health system and wider public.

BAME organisations were going around door to door, giving hand sanitisers, giving masks to everybody [ ]. And it was the BAME community that was the most active during COVID delivering medication, delivering food to houses, doing the shopping. [ ] Nobody gave credit to that. Nobody talks about the good work that the BAME community has done. (1020, community participant)

A number of community and VCFSE sector participants highlighted the work done at the community level, by either themselves or other networks to support local communities. This included providing support packages, running errands for vulnerable community members, cooking and food shopping services, a helpline and communication networks for local communities, and online wellbeing and support groups.

We might not have been in hospital, but we were frontline workers in the community. (1028, community participant)

Support was provided by formal VCFSE organisations and by smaller, sometimes informal, community networks and channels, in which support mechanisms included mental health support and wellbeing focused communications to combat loneliness and boost wellbeing. This was often focused around outreach and the provision of community-based support to the most marginalised and vulnerable groups that had been disproportionately impacted during the pandemic, e.g. recently settled refugees and asylum seekers, older individuals.

We have an Iranian group in Salford…And one of them spotted this young woman in the queue and she thought she looked Iranian, you know….anyway she started a conversation, and this person had been an asylum seeker at the beginning of the pandemic and had been in a detention centre during the pandemic. And then, finally got their leave to remain and then were just basically dumped in Salford. [ ] just having that friendly face and someone was trying to start that conversation, she was able to be linked into this group of women who support other refugees and asylum seekers from the Middle East. (2014, VCFSE participant)

### Community led support to COVID-19 vaccination delivery

The VCFSE sector and community networks also played a crucial part in supporting the COVID-19 vaccine delivery. Community, VCFSE and system-sector participants recognised the unique role that the VCFSE sector had played in reaching diverse communities and sections of communities not reached by the mainstream vaccination programme. For example, VCFSE groups aided vaccine delivery by helping run vaccine ‘pop-up’ sites in community spaces including mosques and other religious sites, children’s centres, and local specialist charities (e.g.: refugee and sex worker charities).

The use of community ‘champions’ and community ‘connectors’ to convey messaging around the vaccination drive were deemed especially vital in this regard. Trusted members of communities (e.g. community leaders) who had crucial pre-existing communication channels were able to effectively interact with different parts of communities to advocate for the vaccine and address misinformation. Situated within communities themselves, these ‘champions’ held established trust within communities, allowing conversations surrounding the vaccine to be held on the basis of shared experiences, honesty, openness, compassion and understanding.

So, as with any ethnic minority community, unless you’re part of it, it’s almost impossible to completely dig out all its norms and its very, very fine distinctions…[ ] what is acceptable, what is not acceptable[ ]? Unless you’re part of it, or you’ve really immersed yourself in the culture for decades, it’s almost impossible to get it (2015, VCFSE participant)

One of the strongest approaches that you can take to increase uptake in any community, whether it be pregnant women or a faith group or a geographical area or a cultural group, is that if you’ve got a representative from that community leading on and advocating for the vaccine, you’re going to have the best impact (2011, health and care system stakeholder participant).

unless Imams or significant people in the community were coming out for them and saying, it’s absolutely fine, it’s safe, and culturally it’s the right thing to do, there was a bit of uncertainty there (2010, health and care system stakeholder participant).

Health and care system stakeholders also emphasised the importance of “community ownership” of vaccination approaches, and of system responsiveness to identified needs and priorities at the community level. Health and care system stakeholders recognised that they were able to utilise community links to have better on-the-ground knowledge, provided in real time, to supplement locally held data to inform targeted efforts to boost uptake. This included council led initiatives including door-knocking with council staff, local health improvement practitioners, and VCFSE representatives working together to provide information about vaccine clinics and register people for vaccine appointments.

if messages went out and they didn’t land right they [the VCFSE sector] could be the first people [that] would hear about that and they could feed that back to us. [ ]….we were able to regularly go to them and say, look from a geographical perspective we can see these key areas…[ ] the people aren’t coming for vaccinations, [ ] what more can you tell us. Or, we can say, from these ethnicities in this area we’re not getting the numbers, what more can you tell us. And when we’ve fed them that intelligence then they could then use that to go and gain further insight for us, so they were a kind of, key mechanism (2010, health and care system participant).

### Operational and logistical barriers to community-based pandemic responses: challenges faced by the voluntary and community sector

VCFSE sector and health and care system stakeholder participants reported significant logistical barriers to partnership working to support communities during the pandemic. Barriers included red tape and bureaucracy, which delayed responses to communities’ health and wellbeing needs.

whilst we were buying masks and hand sanitisers and going door to door, [ ] the council were still getting their paperwork in order, their policies in order, it was meeting after meeting. It took them seven to eight weeks for them to say [ ] we’ve got masks, would you like to help dish them out. (1029, VCFSE participant)

VCFSE and health and care system participants also raised challenges with respect to the VCFSE sector supporting the vaccination programme. This resulted in frustration amongst both VCFSE and health and care system participants who recognised the value of these community-based approaches.

The time that trickles through to the council and the time that the council turn around and say all right, we’ll actually let you do it was weeks later, and the community is turning round to us and saying to us well, what’s going on? We don’t like being messed around like this… (2008, VCFSE participant).

Participants highlighted the numerous health-related bodies with various roles which comprise a complex system for VCFSE partners to navigate, in part due to organisational and cultural clashes. Frustration was felt by both VCFSE and health and care system stakeholder participants (from local councils) in this respect. One VCFSE participant discussing the vaccine rollout noted:

We hit dead end after dead end within the council and there was literally very little response….You’ve got so many departments within this massive organisation called the council…[ ].it’s very difficult to navigate all that and deal with all that bureaucracy… (2008, VCFSE participant).

Broader institutional and organisational barriers to VCFSE support were identified, where cultural clashes between differing values and ways of working emerged, including ethos surrounding risk aversion and the system-level commitment to privilege value-for-money during the vaccination rollout. More practical issues around information governance and training were also raised as barriers to collaborative working.

I don’t think that they understand the power of community and the way community works. I don’t think that at a governmental level they understand what it means to penetrate into a community and actually understand what needs to be done to help a community…[ ] If they did and they had better links and ties into understanding that and helping that then we likely wouldn’t have had so many hurdles to get through (2008, VCFSE participant).

….in terms of public money, this is a public programme, we need to get value for the public pound. So we’re saying to [VCFSE organisation], how much is it going to cost? And [VCFSE organisation] are like, well, we don’t really know, until we deliver it. And we’re like, well, we can’t really approve it, until we know what it’s going to cost…. (2006, health and care system stakeholder participant)

Overall, these issues surmounted to difficulties of power-sharing between public sector organisations and VCFSEs during a time of rapid response to a public health crisis, political, institutional, and other external pressures. This was echoed amongst VCFSE and health and care system stakeholder participants, where frustration towards this was felt from both sides.

the public sector [ ] need to get better at letting go of some of the control. So even still, after I said, so many times, [VCFSE organisation] are delivering this, [VCFSE organisation] are doing everything, [ ] I still got the comms team going, are we doing a leaflet? No, [VCFSE organisation] are doing it, this is a [VCFSE organisation] programme, this isn’t a Council programme. (2006, local authority participant)

it is difficult sometimes working with organisations, I find myself very much stuck in the middle sometimes [ ] I engage with [community groups] and ask them how best we do it and then we put things in place that they’ve asked for, and then they’ve told us it’s not working why have you done it like that. [ ] I think it’s acknowledgement to do it right, it takes time, and it takes effort, it takes resource. (2010, local authority participant)

Health and care system stakeholders also highlighted the importance of accessibility and localised vaccination hubs to reach different parts of diverse local communities e.g. sites in local mosques and sites near local supermarkets to reach different demographics. For instance, having mobile vaccination sites to reduce accessibility barriers, alongside dialogue-based initiatives to answer questions and respond to concerns from local communities about the vaccine, with the view to building trust without explicit pressure to receive the vaccine. Describing their efforts to engage with a member of the local community over the vaccine, two local health and care system stakeholders detailed the following example of how localised, communication-based approaches were deemed successful:

She came to the clinic and there were a lot of tears. It was very emotional. She’d been through a very difficult journey and had got pregnant by IVF, so it was a big decision for her, a big risk that she thought she was taking. Whether she took the vaccine or not, it felt like a risk to her, [ ] we were able to sit down and talk to her. We had some peers there. So we had other pregnant women there who’d had the vaccine, that were able to give her some confidence. We had the specialist multicultural midwife there, [ ] And we literally just sat and drank coffee with her and let her talk and she ended up agreeing to have the vaccine [ ] (2011, system-level stakeholder).

…And the feedback from that lady was amazing. A couple of weeks ago I contacted her to make sure she was going to come down for her booster and she was just so grateful. [ ] she’d had backlash from her family and people within her community for taking up the vaccine and they still thought it was a massive risk. But she had no doubts that she’d done absolutely the right thing… (2012, system-level stakeholder).

### Learning from the pandemic response in GM: trust building and harnessing community assets

Taking these findings from health and care system stakeholders, community and VCFSE participants, several learning points were identified.

In terms of vaccine delivery, some health and care system stakeholder participants reflected the need for more joined-up ways of working, across existing services and amongst VCFSE partners, to ensure efficiency and maximise uptake by embedding the vaccination programmes into other health services. For example, offering vaccination through health visiting or health checks, or offering COVID-19 vaccine boosters and flu vaccinations in single visits at care homes. These settings could also provide opportunities for dialogue with local communities where there is pushback against vaccination. Another health and care system stakeholder identified the need for greater joined up delivery of services; utilising the VCFSE sector to deliver multiple services simultaneously, including the vaccine, to improve vaccine uptake and access to other healthcare services:

the sex worker clinic is a good example of that. [ ] People were coming in for another reason, to get their health check and to get their support from the advisors there at that voluntary organisation, [ ]…if there’s a multiple purpose at the site, for people to attend, you can start to engage them in the conversation and then take the opportunity and vaccinate them. So I’m really interested in looking at that a little bit more, about how that can help to increase uptake. (2011, health and care system stakeholder participant)

A VCFSE participant suggested using educational settings such as schools as a channel to disseminate public health and vaccine-related information, as trusted settings which have wide-reach to many different communities.

A number of health and care system stakeholders, VCFSE and community participants noted that long-term, continuous, meaningful engagement is crucial to build longer-term trust between institutions and communities, and to improve the efficacy of public health measures. It was felt that more concentrated efforts were required from the NHS and other statutory organisations to reach the most marginalised and minoritised communities, for example through door-knocking and welfare calls. Participants highlighted that this was required not solely at times of public health crises, but as part of continued engagement efforts, in order to adequately engage with the most marginalised groups and effectively build long-term trust. This may be done most effectively by building on existing links to marginalised communities, for example using education liaison staff to understand traveller communities’ perspectives on the vaccine.

proactive engagement with communities both locally and nationally to say, [the health system] are looking at this, what’s people’s thoughts, views, you know, is there any issues with this, what more can we do, what do you need to know to make an informed decision. This is what we were thinking of, how would this land…I think we could learn by, [ ] doing that insight work, spending more time working with communities at a kind of, national, regional, and local level (2010, health and care system stakeholder participant).

[the health system] could have engaged better with communities, I think bringing them in at the beginning. So, having them sat around the table, representatives from different groups, understanding how to engage with them from the very beginning…I think they could have used the data very very early on to inform who were engaging. We didn’t quite get it right at the beginning, we didn’t link the public health data teams with the comms and engagement teams (2013, health and care system stakeholder participant).

The tone of communications was also seen to be important. One health and care system stakeholder participant noted that the strategy of pushing communications and public health messaging aimed at behavioural change did not achieve the desired effect as these did not engage effectively with the communities to alleviate or address key concerns about the vaccine. These were deemed less successful than starting from a place of understanding and openness to generate constructive dialogue which could foster trust and respect.

There was also more specific learning identified in terms of collaboration between public sector institutions, VCFSEs and community links, with this seen as vital to build strong, long-term relationships between sectors based on trust and mutual respect. This should also involve working to share knowledge between sectors in real-time.

Health and care system stakeholder and VCFSE participants both suggested a failure to further develop partnerships fostered during the pandemic would be a lost opportunity that could potentially create distrust and additional barriers between communities, VCFSEs and public organisations, perhaps further marginalising seldom-heard groups.

we need to find ways which we have ongoing engagement, and I think it needs to be more informal. People don’t want to be just constantly asked and asked and asked (2010, health and care system stakeholder participant).

a network of just sharing information and insight, rather than just engaging when you’ve got something specific to engage about. (2010, health and care system stakeholder participant)

We were then thinking to ourselves, well, maybe we shouldn’t be doing this. If it’s going to cause us damage, if the council can’t work with us properly maybe we just shouldn’t do it. We’ve got to weigh up. We don’t want to lose our trust within the community (2008, VCFSE participant).

In terms of dynamics and working arrangements between sectors, participants thought it important to allow community organisations and VCFSEs to lead on their areas of speciality, e.g.: community organisations leading on outreach and communications within and to communities. This relates to the identified need of pursuing adaptable and flexible approaches to vaccine delivery. Moreover, there is a need to allow more joined-up decision-making between the health system and VCFSEs to ensure better use of local intelligence and improved planning.

## Discussion & policy implications

### Unmet need and the role of communities during the pandemic

Our findings clearly demonstrate that local communities were not supported sufficiently by mainstream services during the COVID-19 pandemic. This in turn led to frustration, fear and loss of faith in the healthcare system as a whole, evidenced also in responses to the COVID-19 vaccination programme in which distrust results from wider experiences of historical marginalisation and structural inequalities [14]. In the absence of mainstream service support, our findings demonstrate how VCFSE organisations and community networks mobilised to support local communities to fulfil unmet health, social care, and wellbeing needs. This supports emerging evidence from across England which finds that the VCFSE sector played a key role in supporting communities during the pandemic [6, 8, 25].

The importance of community-based, localised approaches, community-led and community owned initiatives, ‘community champions’ and community connectors’ were also highlighted as crucial to the success of the COVID-19 vaccination drive. Participants noted that community-led approaches were uniquely positioned to reach some communities when mainstream approaches were unsuccessful. This is echoed in existing literature, where the role of localised community responses was deemed important to reach marginalised groups, as part of the wider pandemic response [26].

### Operational and logistical barriers

Operational and logistical barriers created dissonance between communities and the system. These barriers included difficulties with decision-making and power-sharing between VCFSE and commissioning or clinical organisations, organisational cultural clashes, red-tape and bureaucracy, and complex systems and power structures to navigate. This builds on existing evidence of barriers to partnership working during the pandemic, including cultural clashes and bureaucracy/red tape [5, 27]. The VCFSE sector also suffered from the closure of services, and reduced funding and resources due to increased demand for services and needing to adapt service provision [8].

These factors hindered collaborative working and created risk for VCFSEs, including putting tension on relationships with local communities resulting from delays implementing services. In most VCFSE-health system partnerships, participants noted that power is generally held by the health system partner, but reputational risk and additional resource-based costs lie with VCFSE partners. Supporting capacity building and workforce resource within the voluntary sector will strengthen this [28].

Inadequate processes to establish collaborative working enhance distrust between the health system and VCFSE sector, which in turn enhances difficulties for collaborative working. Trust is an important factor in how the system interacts with VCFSEs, with a lack of trust leading to further bottlenecks in VCFSE activities [29]. Alongside this, is the need for greater health system appreciation for the VCFSE sector, with VSCE partners reporting they faced greater scrutiny and more arduous administrative processes than private sector partners [2, 29].

### Learning from the pandemic: service prioritisation

All sectors of the health and care system face pressures from resource shortages, internal and external targets [30, 31]. This is often linked to drives to increase the value-for-money of services, but key questions remain as to how to assimilate the goals of achieving health equity within value-for-money objectives [32]. To this end, prioritising value-for-money may come at odds with reducing health inequities. For example, during the rollout of the vaccination programme, additional resources and innovative approaches were required to reach marginalised communities [33, 34]. This is supported by emerging evidence from England and internationally that efforts to drive vaccination uptake and reduce inequities in uptake amongst marginalised populations require significant resources and a breadth of approaches to maximise uptake [34]. Our findings suggest that changes in vaccine uptake were smaller and slower to be realised in these populations, resulting in a “slow burn” in terms of demonstrating quantifiable outcomes. Given the NHS principles of equity [10, 35], reaching these groups should remain a public health priority, and failure to prioritise these groups may incur greater long-term financial costs resulting from greater health service needs. Our findings support that challenging entrenched attitudes and frameworks for how success is measured and adapting structures to better incentivise targeted interventions for marginalised or high-risk groups is essential to prioritising addressing unmet needs amongst marginalised communities.

### The changing commissioning landscape

The development of ICSs and place-based partnerships has changed how health and care services are commissioned. National guidance encourages health and care leaders to include VCFSE organisations in partnership arrangements and embed them into service delivery [12], with ‘alliance models’ between ICSs and the VCFSE sector [36] established in certain regions (see for example [37]. However, this rests on “a partnership of the willing” [37] between ICS partners and VCFSE sector players, and concrete guidance for achieving collaborative working in practice, is lacking. As the findings in this paper point to, evolving decision-making processes may add to resource burdens for VCFSE organisations. Traditional health and care partners such as the NHS and local authorities should consider how their ways of working may need to change to foster full VCFSE inclusion on an equal standing, otherwise only the VCFSE stakeholders with sufficient capacity and resource may be able to be meaningfully involved.

### Creating a VCFSE-accessible health and care system

In terms of fostering relationships between different sectors, participants acknowledged that pre-pandemic efforts to engage communities and community networks and VCFSEs were insufficient, with more meaningful, well-resourced engagement required going forward. It was also identified by participants the importance of avoiding tokenistic involvement of the VCFSE sector, which may be counter-productive for developing meaningful long-term partnerships. More equal relationships between statutory and VCFSE sectors are needed to foster improved collaborative working [5, 38], and this is identified already at the GM level [28]. Central to this is actioned principles of co-design, including power-sharing, community ownership and trust. In order for co-design strategies to be successful, recognition of the role of the VCFSE sector and their ownership of approaches must be championed within co-design strategies and the enactment of co-designed activities.

Relatedly, greater trust of the VCFSE sector to deliver services effectively and efficiently is needed from health and social care decision-makers to ensure that funding compliance measures and processes are proportionate and not overly burdensome, to avoid funding bottlenecks which in turn impact service delivery [2]. Currently at the national level, VCFSE applicants typically only become aware of funding through existing networks, leaving less-connected organisations to find out ‘by chance’, thereby limiting reach amongst other organisations [2]. This may be especially true for smaller or ad-hoc VCFSE networks and groups. Our findings support that bottlenecks to applying for funding should be removed, and more streamlined processes for accessing funding championed [2].

Our findings also suggest that health systems should engage with the full breadth of the VCFSE sector, creating space for the involvement of smaller scale and less formal organisations as partners. Sharing of best practice and advice for adapting to local contexts should be promoted, alongside evaluation of collaborative models.

Finally, the pandemic period saw unprecedented state-sponsored investment into the VCFSE sector [29]. Within the GM context, this funding enabled VCFSEs to develop organisational capacity and systems, develop new partnerships, and better respond to the (unmet) needs of local communities [39]. Currently there are no clear plans to maintain this investment, but sustained inter-sector partnership working will require continued investment in the VCFSE sector.

### Strengths & limitations

There are two main limitations to this study. Firstly, whilst the study achieved diversity in its sample, we could not achieve representation across all marginalised communities and therefore could not cover the experiences of all marginalised communities in-depth. As such, whilst the analyses provides valuable insights, such insights may not be transferrable and do not reflect all communities in GM. Secondly, whilst other studies focused on multiple city-regions or areas, our study is limited to the city region of GM. However, this focus provides an in-depth analysis on one region, and, as we discuss in the framing of the paper, we contend that the analysis presented in this paper serves as an exemplar to explore further at the national and international level. It should also be noted that co-design approaches are inevitably time and resource-heavy, and this was challenging in the context of this study, as local stakeholders wanted timely insights to inform the vaccination programme. However, one of the key strengths of our participatory approach was that this enabled a direct connection with the experiences of communities as relevant to the research, in order to shape the research questions, as well as the design and conduct of the study.

## Conclusions

Overall, the contribution of the VCFSE sector during the pandemic is clear, with significant support provided in respect to community health and wellbeing and vaccination delivery. Nevertheless, there remains much to learn from the pandemic period, with the potential to harness capacity to tackle inequalities and build trust through shared learning and greater collaborative working. Maintaining an environment in which VCFSE partners are under-recognised, under-valued, and seemingly face further bureaucratic barriers will only exacerbate issues to collaborative working. There are also significant questions around systemic issues and sustainability, which must be addressed to overcome existing barriers to collaborative working between sectors. For instance, our findings identify the importance of flexibility and adaptability, in ongoing and future service delivery. Where this is not pursued this may not only impact service delivery but also create roadblocks to collaboration between sectors, creating divisions between entities whilst ultimately trying to effect change on similar goals (i.e. improved population health). ICS–VCFSE Alliances and community connectors may be a mechanism to promote this, but clear, actionable guidance will be required to translate rhetoric to real-world progress.

### Electronic supplementary material

Below is the link to the electronic supplementary material.

Supplementary Material 1

Supplementary Material 2

Supplementary Material 3

Supplementary Material 4

Supplementary Material 5

Supplementary Material 6

## Data Availability

Data for this research data will not be made publicly available as individual privacy could be compromised. Please contact Stephanie Gillibrand (stephanie.gillibrand@manchester.ac.uk) for further information.

## Abbreviations

CCGs: Clinical Commissioning Groups
CRAG: Community Research Advisory Group
GM: Greater Manchester
ICSs: Integrated Care Systems
VCFSE: Voluntary, Community and Social Enterprise

## Footnotes

1 10 GM is an umbrella group which seeks to represent the VCSE sector in GM. More information is available here: https://10gm.org.uk/.

2 These themes are explored in a related paper by Gillibrand et al. [14].

3 Topic guides are provided as supplementary material.

4 Distrust was also raised in relation to fear and anxiety in NHS settings, and this is discussed in detail in a related paper from this study by Gillibrand et al. [14].
