# Supplementary material for: “We might not have been in hospital, but we were frontline workers in the community”: a qualitative study exploring unmet need and local community-based responses for marginalised groups in Greater Manchester during the COVID-19 pandemic
Source: BMC Health Serv Res. 2024 May 13;24:621. doi: 10.1186/s12913-024-10921-4 (PMC11092215; doi:10.1186/s12913-024-10921-4)
Supplement: Supplementary file 2 — Supplementary Material 2 [file 12913_2024_10921_MOESM2_ESM.docx]

**Understanding community experiences during COVID-19 and attitudes towards the COVID-19 vaccine**

**Semi-structured interview schedule**

Provide a brief explanation of what we want to talk about, for example:

*We want to ask you about your experiences during the COVID-19 pandemic, including views and experiences of the Covid-19 vaccination programme.*

*We will also discuss experiences of the healthcare system more broadly.*

*We are also interested to hear your thoughts about how those in the public health, research and policy setting can better serve communities.*

*All views and opinions will be respected and are encouraged.*

*Example questions*

- Begin the interview with an open question connected to recent experience, such as:

1. *To begin with, which community groups are you most involved with and in which area?*
2. *I would like you to think about these communities, and other community groups you may have been involved with during the pandemic. Thinking about health and the healthcare system, in your opinion, what have the community(ies) experienced during the pandemic? How has the pandemic impacted different groups in your community?*
3. *In your opinion, what are the community’s thoughts towards the vaccine been? Have different members of your community held different views about the vaccine? What are the main reasons for taking up the vaccine, what are the main concerns towards the vaccine?*
4. *Have these views changed since the vaccine started to be rolled out?*
5. *In your opinion, what could the health system (academic researchers, healthcare professionals* *and those making decisions) do to address the community’s concerns about the vaccine? Which local or national policies or strategies do you think were affective and which were ineffective?*
6. *From our previous public engagement work, mistrust – of information, the government and NHS, and pharmaceutical companies, were mentioned as important to the public. Is this something you have encountered amongst the community? What are the main reasons behind this? How does this vary across different groups in your community?*
7. *What could the health system (academic researchers, healthcare professionals and those making decisions) do to address public mistrust? How could the health system have better engaged with the public and communities during the pandemic?*
