# Supplementary material for: “We might not have been in hospital, but we were frontline workers in the community”: a qualitative study exploring unmet need and local community-based responses for marginalised groups in Greater Manchester during the COVID-19 pandemic
Source: BMC Health Serv Res. 2024 May 13;24:621. doi: 10.1186/s12913-024-10921-4 (PMC11092215; doi:10.1186/s12913-024-10921-4)
Supplement: Supplementary file 4 — Supplementary Material 4 [file 12913_2024_10921_MOESM4_ESM.docx]

**Understanding community experiences during COVID-19 and attitudes towards the COVID-19 vaccine**

**Semi-structured topic guide for community and public focus groups**

*We want to ask you about your experiences during the COVID-19 pandemic, including views and experiences of the Covid-19 vaccination programme.*

*We will also discuss experiences of the healthcare system more broadly.*

*We are also interested to hear your thoughts about how those in the public health, research and policy setting can better serve communities.*

*All views and opinions will be respected and are encouraged.*

**Example questions**

1. *Thinking about your health and your experiences with healthcare professionals or the healthcare system, what have been your experiences during the COVID-19 pandemic?*
2. *Are these experiences different to your experiences before the COVID-19 pandemic?*
3. *How has COVID-19 impacted your community/age-group in Greater Manchester?*
4. *What are your thoughts towards the COVID-19 vaccine? What are the main reasons to have the vaccine, or deciding not to have the vaccine?*
5. *Have these views changed since the vaccine started to be rolled out? Have people around you held similar views to you, and have their views changed over time?*
6. *Have social networks, your community, friends, peers and family shaped your views on COVID-19 and the vaccines?*
7. *In your opinion, what could the health system (**academic researchers, healthcare professionals and those making decisions) do to address the public’s concerns about the vaccine?* *Which local or national policies or strategies do you think were affective and which were ineffective?*
8. *From our previous public engagement work, mistrust – of information, the government and NHS, and pharmaceutical companies, were mentioned as important to the public. What are your thought on this?*
9. *What could the health system (academic researchers, healthcare professionals and those making decisions) do to address public mistrust? How could the health system have better engaged with the public and communities during the pandemic?*
10. *What are your views about a) sharing and using b) generating new forms of anonymous personal health information (i.e. that is not identifiable) for researching how COVID-19 impacts different groups differently, and for researching the COVID-19 vaccine?*
