# Supplementary material for: “We might not have been in hospital, but we were frontline workers in the community”: a qualitative study exploring unmet need and local community-based responses for marginalised groups in Greater Manchester during the COVID-19 pandemic
Source: BMC Health Serv Res. 2024 May 13;24:621. doi: 10.1186/s12913-024-10921-4 (PMC11092215; doi:10.1186/s12913-024-10921-4)
Supplement: Supplementary file 5 — Supplementary Material 5 [file 12913_2024_10921_MOESM5_ESM.docx]

| **Consolidated criteria for reporting qualitative studies (COREQ): 32-item checklist**  ***adapted from Tong et al 2007** | |
| --- | --- |
| 1. Interviewer*/*facilitator | Details are provided but redacted currently.  Specified on page 11 |
| 1. Credentials | Details are provided in author information |
| 1. Occupation | Details are provided in author information |
| 1. Gender | Details are provided in author information |
| 1. Experience and training | Some of the CRAG had prior connections with VCSE groups who were used to recruit from, and some of th research team had prior connections to CRAG members. This is detailed on page 11 |
| 1. Relationship established | No direct relationship with participants was held prior to the study. |
| 1. Participant knowledge of the interviewer | A short introduction to the study and interviewer was provided at the start of the interviews. |
| 1. Interviewer characteristics | Positionality of the researchers is provided on page 11 |
| 1. Methodological orientation and Theory | An adapted thematic framework approach was used, this is stated on page 14-15 |
| 1. Sampling | Purposive sampling was undertaken, this is detail on page 12-13 |
| 1. Method of approach | Participants were approached by connections with pre-established links to local community organisations. This is detailed on page 12. |
| 1. Sample size | Total of 35 participants |
| 1. Non-participation | We did not routinely track non participation or drop outs. |
| 1. Setting of data collection | Data was collected remotely due to the pandemic. This is stated on page 11 |
| 1. Presence of non-participants | In two of the focus groups a CRAG member was present who helped to facilitate. This is detailed on page 11 |
| 1. Description of sample | Characteristics of the sample are provided in Table 1 on page |
| 1. Interview guide | Semi structured interviews were undertaken. Topic guides are provided as supplementary material . |
| 1. Repeat interviews | No repeat interviews were undertaken |
| 1. Audio*/*visual recording | Audio recording was used for transcription purposes and stored securely on the University’s system inline with the University’s SOP |
| 1. Field notes | Field notes were made during and after the interviews. |
| 1. Duration | Duration data has been added on page 11 |
| 1. Data saturation | Data saturation was not explicitly discussed rather was guided by diversity achieved in sampling. |
| 1. Transcripts returned to participants | No, but transcripts were checked by the researchers with the original audio files |
| 1. Number of data coders | 3 members of the research team coded the transcripts. Details of who coded the data is provided on page 14-15 |
| 1. Description of the coding tree Did authors provide a description of the coding tree? | Details of the themes/sub themes are provided on page 15 |
| 1. Derivation of themes | Themes were derived from the data |
| 1. Software | n/a |
| 1. Participant checking | A CRAG member is a co-author on the paper. The paper will be disseminated to all participants once published. |
| 1. Quotations presented | Yes quotes are provided with a participant identifier number throughout the Results section |
| 1. Data and ﬁndings consistent | Findings are consistent with the data. |
| 1. Clarity of major themes | Themes are provided in the Results section |
| 1. Clarity of minor themes | Initial set of themes are detailed in the Methods section on page |
