# Supplementary material for: “We might not have been in hospital, but we were frontline workers in the community”: a qualitative study exploring unmet need and local community-based responses for marginalised groups in Greater Manchester during the COVID-19 pandemic
Source: BMC Health Serv Res. 2024 May 13;24:621. doi: 10.1186/s12913-024-10921-4 (PMC11092215; doi:10.1186/s12913-024-10921-4)
Supplement: Supplementary file 6 — Supplementary Material 6 [file 12913_2024_10921_MOESM6_ESM.docx]

**Initial themes & sub-themes**

| **Theme 1 Community responses & experiences** | **Theme 2 Access to healthcare services** | **Theme 3 Operational and logistical factors around pandemic response** |
| --- | --- | --- |
| - Value of community / community approaches (community organisations, comm leaders/champions) - Variation within communities - Community/family tension - Tension/divisions between communities - Community resilience - Collective trauma (racial, grief) - Gendered responses (caring, support) | - Lack of access to specialist services (e.g. mental health, coma support) - Lack of access to routine and general health services (e.g. GP) - Experiences of care during pandemic | - Value of community approaches (community champions/organisations - Models of delivery of rollout, - Logistical challenges, - Commissioning challenges - Organisational/institutional barriers (vaccine rollout; support packages) - Learning? Flexible approaches to delivery? |

*only initial themes relevant to below are includes in the table above for clarity

**Final set of themes**

| **Theme 1: Unmet needs of local communities during pandemic** | **Theme 2 value of community led-approaches during pandemic** | **Theme 3 Operational and logistical barriers to community-based pandemic responses** |
| --- | --- | --- |
| - Lack of access to specialist services (e.g. mental health, coma support) - Lack of access to routine and general health services (e.g. GP) - Experiences of care during pandemic | - Value of community / community approaches – wider pandemic response - **Value of community approaches (community champions/organisations for vaccine rollout** | - Models of delivery of rollout, - Logistical challenges, - Commissioning challenges - Organisational/institutional barriers (vaccine rollout; support packages) - **Learning from the pandemic response** |

*sub-themes which appear as separate headings in the results section are in highlighted in bold
